# Poor Prognoses of Young Hepatocellular Carcinoma Patients with Microvascular Invasion: A Propensity Score Matching Cohort Study

**DOI:** 10.1155/2020/4691425

**Published:** 2020-02-14

**Authors:** Lian Li, Liangliang Xu, Tianfu Wen, Hong Wu, Wentao Wang, Jiayin Yang, Zheyu Chen, Yonggang Wei, Mingqing Xu, Bo Li, Ming Zhang

**Affiliations:** ^1^Department of Liver Surgery, West China Hospital, Sichuan University, Chengdu 610041, China; ^2^Department of General Surgery, Mianzhu Hospital of West China Hospital, Sichuan University, Mianzhu 618200, China

## Abstract

The relationship between age and the prognosis of patients with hepatocellular carcinoma (HCC) has been widely investigated. However, few studies have focused on the influence of patient age on the prognosis of HCC with microvascular invasion (MVI). Patients with histologically confirmed HCC with MVI who underwent hepatectomy between 2008 and 2016 were retrospectively enrolled in this study and allocated to younger (young group) and older age groups (old group) according to age< or ≥60 years. A propensity score matching analysis was performed, and prognostic factors evaluated by Kaplan–Meier curves and Cox proportional hazards regression. Intraoperative and postoperative characteristics were compared between the two groups. A total of 374 patients were enrolled in this study. There were 84 patients in each group after a 1 : 1 propensity score matching analysis. The rates of both disease-free survival (DFS) and overall survival (OS) differed significantly between the age groups. By univariate and multivariate analyses, age < 60 years was significantly associated with DFS (hazard ratio, 1.590; 95% CI, 1.135–2.228) and OS (hazard ratio, 1.837; 95% CI, 1.259–2.680). There were no significant differences in intraoperative or postoperative characteristics between the two age groups. In patients with histologically confirmed HCC with MVI, the prognosis is poorer for those aged younger than 60 years than for those aged 60 years or older. Hepatectomy can be safely performed in selected older patients.

## 1. Introduction

Liver cancer has recently been reported to have the sixth highest incidence and to be the fourth leading cause of cancer death among all neoplasms worldwide [1]. Hepatocellular carcinoma (HCC) is the most prevalent type of liver cancer, comprising 75% to 85% of all cases [2]. Hepatectomy remains one of the most effective treatment strategies for selected patients with HCC [1, 3]. However, poor prognosis is the biggest obstacle to the treatment of HCCs [4]. Microvascular invasion (MVI) is the risk factor that is most closely related to the prognosis of patients with HCC [5, 6] and has therefore been widely studied in recent years [7–10].

Tumor-related problems in older individuals have recently aroused great concern [11], health problems in this age group being increasingly prominent with the aging of populations worldwide. Over the past two decades, the age-adjusted morbidity of HCC has increased because of aging and population growth [2, 12]. It has been reported that age is not associated with prognosis in patients with HCC who have undergone hepatectomy [13]. However, another study found that the survival of older individuals after liver resection for HCC is poor, despite the fact that the estimated relative survival suggests that hepatectomy can benefit these patients [14]. Moreover, older and younger patients with HCC are often treated differently [15].

In terms of the established correlation between MVI and prognosis of HCC and the possible difference in prognosis between younger and older patients, few studies have compared the outcomes of patients with MVI according to age group. Therefore, the aim of this study was to investigate the differences in prognosis between younger and older patients with HCC and MVI (HCC-MVI).

## 2. Materials and Methods

### 2.1. Patients

Data of 714 patients with HCC-MVI who had undergone hepatectomy in our department from January 2008 to November 2016 were retrospectively collected. All enrolled patients had pathologically confirmed HCC-MVI. The definition of MVI was microscopic tumor invasion identified in the portal or hepatic vein of the surrounding liver tissue, contiguous with the tumor. In accordance with the definition of the World Health Organization, older patients were defined as those aged 60 years or older [16]. Thus, the patients were allocated to older (age greater than or equal to 60 years) and younger age groups (age less than 60 years) in this study. Our study was approved by the West China Hospital of Sichuan University Biomedical Research Ethics Committee. Because this was a retrospective study of anonymized data, consent to participate was not required. The clinical trial registration number is ChiCTR2000029320.

To avoid bias, 1 : 1 propensity score matching (PSM) between the two cohorts was performed, and 84 patients enrolled into each group. Inclusion criteria comprised of (1) generally good condition without major organ dysfunction, (2) no history of another malignant tumor, (3) pathologically confirmed MVI, and (4) having undergone curative resection.

### 2.2. Design

Patients diagnosed with HCC in accordance with published diagnostic criteria for HCC [17] were allocated to older and younger age groups. To fully assess the patient's general condition before surgery, abdominal enhanced computed tomography (CT) or magnetic resonance imaging, cardiopulmonary function, and serological testing, including hepatitis B surface antigen, alpha-fetoprotein (AFP), gamma-glutamyl transpeptidase (GGT), aspartate aminotransferase, alanine aminotransferase, white blood cell count, lymphocyte count, and total bilirubin were performed. Disease-free survival (DFS) and overall survival (OS) were compared between the two age groups using the Kaplan–Meier method, and significant differences were identified using log-rank analysis. Univariate and multivariate Cox proportional hazards regression analyses were performed to identify significant and independent risk factors for poor prognosis.

### 2.3. Follow-Up

Follow-up ended in November 2016 or at death. DFS was defined as time from hepatectomy to the first detectable recurrence and OS time as time from hepatectomy to death or last follow-up. After the operation, the patient was followed up for the first month and thereafter every three months. Routine blood examination, liver function, serum AFP concentrations, and ultrasonic examinations (ultrasound, contrast-enhanced ultrasound) were routinely performed at each follow-up. If a definite or possible recurrence was detected, further tests, such as abdominal enhanced CT or abdominal enhanced magnetic resonance imaging, were performed and treatment decisions made after the patient's condition had been fully assessed by our multidisciplinary team, which consisted of hepatic surgeons, an oncologist, and a radiologist.

### 2.4. Statistical Analysis

Categorical variables were compared using the *χ*^2^ or Fisher exact test, whereas continuous variables were compared using the Mann–Whitney *U* test. Survival curves of the two study groups were constructed using the Kaplan–Meier method, and the survival curves were compared using the log-rank test. To identify factors independently associated with OS and DFS, univariate analysis was carried out using a Cox proportional hazards stepwise model; the significant (*P* < 0.05) variables were then subjected to stepwise multivariate analysis. To overcome possible selection bias, 1 : 1 PSM between the old group and young group was applied using the nearest-neighbour matching method with a caliper of 0.02 [18]. All analyses were performed using SPSS Statistics version 22.0 for Windows (IBM Corp).

## 3. Results and Discussion

### 3.1. Patient Characteristics

**Figure 1:**
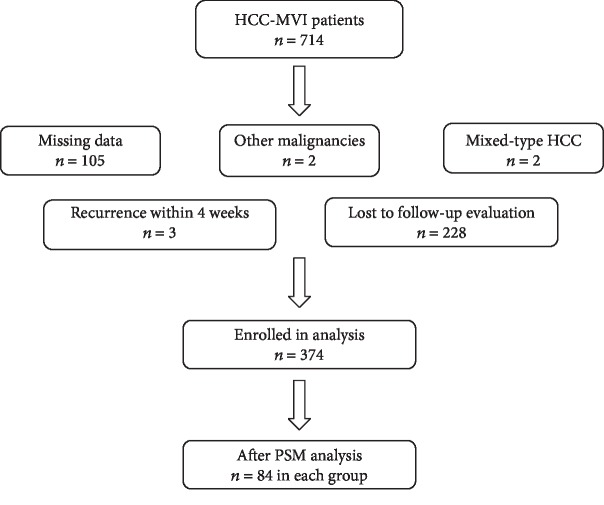
Flow chart of the study participants.

A total of 714 eligible patients were retrospectively identified. The final analysis did not include individuals who were excluded because they were found to have other malignancies during follow-up (*n* = 2), had data missing (*n* = 105), were pathologically confirmed as having mixed-type HCC (*n* = 2), had recurrence within 4 weeks (*n* = 3), or were lost to follow-up (*n* = 228). Finally, 374 patients were included in the analysis (Figure 1). As shown in Table 1, the baseline characteristics of the two age groups differed before the PSM, whereas after the PSM, there were no significant differences between them (Table 2). No significant differences in intraoperative or postoperative characteristics were identified between the two age groups (Table 3).

### 3.2. Survival Analysis

**Figure 2:**
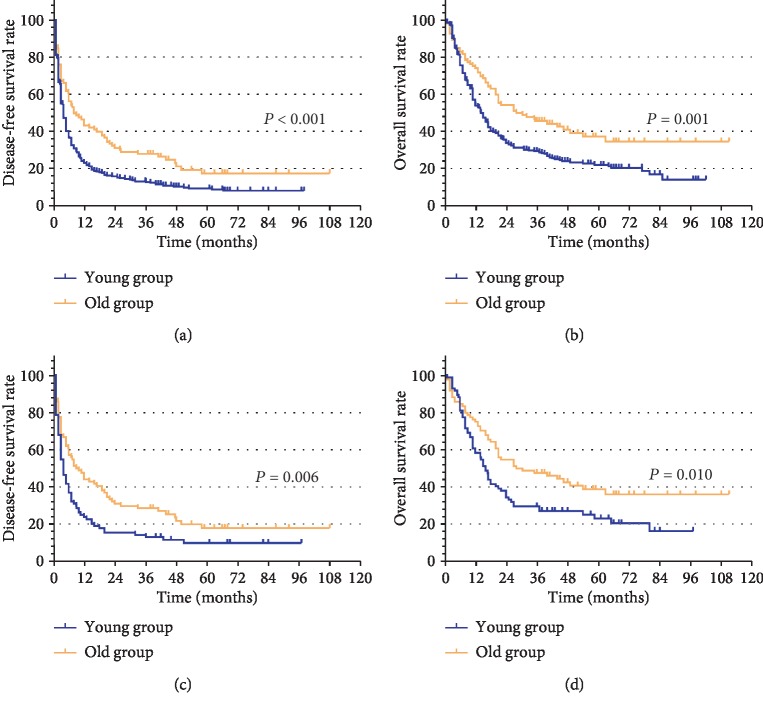
Kaplan–Meier analysis of disease-free survival and overall survival for hepatocellular carcinoma patients with microvascular invasion: (a) disease-free survival for the young group and old group before PSM. (b) Overall survival for the young group and old group before PSM. (c) Disease-free survival for the young group and old group after PSM. (d) Overall survival for the young group and old group after PSM.

Before PSM analysis, the estimated 6-month, 1-year, 2-year, and 5-year recurrence rates in the younger group were 62.8%, 76.4%, 83.7%, and 90.3%, respectively, and in the older group were 43.5%, 56.5%, 68.5%, and 82.2%, respectively (Figure 2(a); *P* < 0.001). The estimated 6-month, 1-year, 2-year, and 5-year OS rates were 75.5%, 54.2%, 33.9%, and 22.2%, respectively, in the younger group and 82.6%, 73.9%, 54.3%, and 37.3%, respectively, in the older group (Figure 2(b); *P* = 0.001). After PSM, the estimated 6-month, 1-year, 2-year, and 5-year recurrence rates were 63.1%, 76.2%, 84.5%, and 90.0%, respectively, in the younger group and, the 42.9%, 56.0%, 69.0%, and 82.1%, respectively, in the older group (Figure 2(c); *P* = 0.006). The estimated 6-month, 1-year, 2-year, and 5-year OS rates were 81.0%, 58.3%, 34.5%, and 23.2%, respectively, in the younger group and 84.5%, 75.0%, 54.8%, and 38.9%, respectively, in the older group (Figure 2(d); *P* = 0.010).

### 3.3. Independent Risk Factors of Prognosis

As shown in Table 4, on univariate analysis, age, postoperative adjuvant transcatheter arterial chemoembolization (TACE), Barcelona Clinic Liver Cancer (BCLC) stage, and lymphocyte (LYM) count were identified as significant risk factors for DFS, and age, BCLC stage, reoperation, giant vascular invasion, and LYM count were identified as significant risk factors for OS. Multivariate analyses revealed that the following factors were significantly associated with DFS: age (hazard ratio, 1.590; 95% CI, 1.135–2.228), postoperative adjuvant TACE (hazard ratio, 1.647; 95% CI, 1.170–2.320), and LYM count (hazard ratio, 0.653; 95% CI, 0.484–0.880). Similarly, multivariate analyses showed that age (hazard ratio, 1.837; 95% CI, 1.259–2.680), reoperation (hazard ratio, 1.647; 95% CI, 1.170–2.320), and LYM count (hazard ratio, 0.592; 95% CI, 0.419–0.838) were associated with OS.

## 4. Discussion

Our study was large, enabling us to perform PSM. There were no statistically significant differences in baseline data between the age groups selected by PSM. We mainly investigated the difference in prognosis between older and younger patients with MVI. Unlike other studies that have reported that age is not a risk factor for prognosis or that older adults have a worse prognosis than younger ones [19, 20], we found that older patients have a better prognosis than younger ones.

The biological behaviour of HCC reportedly differs greatly between younger and older individuals. The reasons for this difference are not fully understood but are likely due to differences in hepatocarcinogenesis [21, 22]. Previous data have indicated that androgen receptor (AR) and phosphoinositide-3 kinase (PI3K) are upregulated and become dominant pathways in tumor tissues with aging [23–25]. Overexpression of AR and PI3K pathways is significantly associated with poor survival [25–28]. This is at variance with the finding in our study that older age is associated with a better survival in patients with HCC.

This discrepancy may be attributable to the fact that our study included only patients with MVI. Some previous studies have reported that younger patients have a worse prognosis, which is consistent with our results; however, those researchers considered that the poorer prognosis is mainly attributable to worse tumor-related indicators, such as larger tumor diameter, later tumor stage, and higher AFP concentration [15 , 29, 30]. In our study, both before and after performing PSM, there was no significant difference in these indicators between the two age groups of patients. We therefore do not believe that the identified prognostic differences are solely due to differences in these factors. MVI status was not considered in the abovementioned studies, despite it reportedly being strongly associated with a poor prognosis [5, 6]. As mentioned earlier, differences in age can be associated with different biological behaviours and some researchers consider that the impact of MVI status on the biological behaviour of tumors varies with age [31, 32]. Thus, differences in age may lead to different biological behaviours of MVI, resulting in different outcomes.

One of the drivers of the increasing incidence of HCC is the progressive aging of the population, which reportedly contributed 16% of the 38% increase in cases from 2006 to 2016 [33]. Some studies have found that older patients are less tolerant than younger patients to surgical resection, which is a complex procedure, and thus may be treated more conservatively than younger patients for the same condition [15, 34]. However, a growing body of research has confirmed that older patients with HCC who undergo resection can achieve outcomes comparable to those of younger patients [13, 14, 23, 35]. This is largely attributable to considerable improvements in postoperative medical care and therefore better control of postoperative complications in older patients. Even individuals older than 75 years have been reported to undergo surgery safely [36]. In addition, in our study data, we found no significant differences in intraoperative or postoperative clinical variables between the two age groups of patients. Therefore, postoperative complications are no longer a barrier to surgery on older patients. Admittedly, the cardiopulmonary function of the older patients in our study was rigorously assessed.

The BCLC classification system for HCCs does not include age or MVI [2]; we believe that more attention to these two factors is justified. Older people tend to receive more conservative treatment than younger individuals for the same condition and thus may miss out on the most effective treatments. The present study provides evidence for treating older persons more actively. In addition, with their poor prognosis, more aggressive preventive measures should be recommended to younger individuals who undergo hepatectomy and have histopathologically confirmed MVI to reduce the likelihood of recurrence.

This study had some limitations. First, it was a retrospective study, albeit including PSM. Randomized controlled trials are needed to confirm our findings. Second, the sample size was relatively small after PSM. Thus, our findings should be verified in larger studies. Future studies should also investigate the correlation of other risk factors, especially those that are well recognized, and age in the prognoses of patients with HCC-MVI.

## 5. Conclusions

In patients with histologically confirmed HCC-MVI, the prognosis of those older than 60 years is superior to that of younger patients. Older individuals can undergo hepatectomy safely, and postoperative complications are no longer a barrier to surgery for them.

## Figures and Tables

**Table 1:** Baseline characteristics of the study participants before PSM.

Variable	Young group	Old group	*P* value
	*n* = 282	*n* = 92	
Gender (male)	250 (88.7%)	78 (84.8%)	0.326
Adjuvant TACE	126 (44.7%)	32 (34.8%)	0.095
Reoperation	29 (10.3%)	5 (5.4%)	0.160
Tumor diameter (cm)	8.1 ± 3.8	7.5 ± 3.4	0.154
Tumor number (single)	209 (74.1%)	67 (72.8%)	0.807
GVI	97 (34.4%)	27 (29.3%)	0.372
Transfusion	43 (15.2%)	12 (13.0%)	0.604
Diabetes	11 (3.9%)	10 (10.9%)	0.012
HBsAg positivity	261 (92.6%)	71 (77.2%)	<0.001
AFP (ng/mL) (IQR)	1210.0 (45.8-1210.0)	324.1 (17.2-1210.0)	0.046
Invading adjacent organs	33 (11.7%)	11 (12.0%)	0.948
Anatomic resection	175 (62.1%)	55 (59.8%)	0.697
Well differentiation	121 (42.9%)	48 (52.2%)	0.121
Invasion of liver capsule	81 (28.7%)	28 (30.4%)	0.754
Satellite nodules	65 (23.0%)	9 (9.8%)	0.006
Lymphatic metastasis	11 (3.9%)	3 (3.3%)	0.779
Cirrhosis	140 (49.6%)	40 (43.5%)	0.304
GGT level (U/L) (IQR)	101.0 (50.0-201.0)	77.5 (43.5-119.8)	0.006
ALT level (U/L) (IQR)	42.0 (30.0-67.0)	36.0 (25.0-59.3)	0.028
AST level (U/L) (IQR)	50.0 (33.8-78.0)	45.5 (31.0-70.8)	0.217
TBIL level (mmol/L)	12.4 ± 1.3	12.0 ± 1.1	0.006
LYM count (10^9^/L) (IQR)	1.4 (1.0-1.8)	1.3 (1.0-1.9)	0.933
WBC count (10^9^/L) (IQR)	5.5 (4.5-6.8)	5.4 (4.3-6.7)	0.928
BCLC staging			0.597
A	48 (17.0%)	15 (16.3%)	
B-C	234 (82.9%)	77 (83.7%)	
Child-Pugh			0.592
A	262 (92.9%)	88 (95.7%)	
B	17 (6.0%)	3 (3.3%)	
C	3 (1.1%)	1 (1.1%)	

Abbreviations: TACE: transcatheter arterial chemoembolization; GVI: giant vascular invasion; HBsAg: hepatitis B surface antigen; AFP: alpha fetoprotein; IQR: interquartile range; GGT: gamma-glutamyl transpeptidase; ALT: alanine aminotransferase; AST: aspartate aminotransferase; TBIL: total bilirubin; LYM: lymphocyte; WBC: white blood cell; BCLC: Barcelona Clinic Liver Cancer.

**Table 2:** Baseline characteristics of the study participants after PSM.

Variable	Young group	Old group	*P* value
	*n* = 84	*n* = 84	
Gender (male)	71 (84.5%)	70 (83.3%)	0.834
Adjuvant TACE	37 (44.0%)	29 (34.5%)	0.206
Reoperation	10 (11.9%)	5 (6.0%)	0.176
Tumor diameter (cm)	7.6 ± 3.6	7.3 ± 3.1	0.583
Tumor number (single)	65 (77.4%)	61 (72.6%)	0.476
GVI	24 (28.6%)	25 (29.8%)	0. 865
Transfusion	8 (9.5%)	10 (11.9%)	0.618
Diabetes	8 (9.5%)	5 (6.0%)	0.386
HBsAg positivity	73 (86.9%)	68 (81.0%)	0.294
AFP (ng/mL) (IQR)	1210.0 (65.4-1210.0)	324.1 (17.2-1210.0)	0.134
Invading adjacent organs	10 (11.9%)	9 (10.7%)	0.808
Anatomic resection	48 (57.1%)	50 (59.5%)	0.754
Well differentiation	33 (39.3%)	42 (50.0%)	0.162
Invasion of liver capsule	22 (26.2%)	25 (29.8%)	0.606
Satellite nodules	11 (13.1%)	9 (10.7%)	0.634
Lymphatic metastasis	4 (4.8%)	3 (3.6%)	0.699
Cirrhosis	40 (47.6%)	37 (44.0%)	0.642
GGT level (U/L) (IQR)	75.5 (43.5-129.5)	67.0 (42.3-119.8)	0.526
ALT level (U/L) (IQR)	43.0 (28.5-66.8)	37.5 (25.0-60.8)	0.124
AST level (U/L) (IQR)	45.5 (33.0-69.5)	47.0 (32.3-71.0)	0.858
TBIL level (mmol/L)	12.0 ± 1.2	12.0 ± 1.1	0.852
LYM count (10^9^/L)	1.5 ± 0.6	1.5 ± 0.6	0.876
WBC count (10^9^/L) (IQR)	5.4 (4.4-6.9)	5.4 (4.3-6.7)	0.878
BCLC stage			0.592
A	18 (21.4%)	13 (15.5%)	
B-C	64 (78.6%)	71 (84.6%)	
Child-Pugh			0.592
A	79 (94.0%)	81 (96.4%)	
B	2 (2.4%)	3 (3.6%)	
C	3 (3.6%)	0 (0.0%)	

Abbreviations: TACE: transcatheter arterial chemoembolization; GVI: giant vascular invasion; AFP: alpha fetoprotein; IQR: interquartile range; GGT: gamma-glutamyl transpeptidase; ALT: alanine aminotransferase; AST: aspartate aminotransferase; TBIL: total bilirubin; LYM: lymphocyte; WBC: white blood cell; BCLC: Barcelona Clinic Liver Cancer.

**Table 3:** Intraoperative and postoperative characteristics of the study participants after PSM.

Variable	Young group	Old group	*P* value
	*n* = 84	*n* = 84	
Intraoperative blood loss (mL) (IQR)	300 (200-437.5)	300 (200-575.5)	0.398
Intraoperative RBC transfusion (U) (EVR)	0 (0-17.5)	0 (0-7)	0.779
Intraoperative plasma transfusion (mL) (EVR)	0 (0-1800)	0 (0-400)	0.680
Postoperative RBC transfusion (U) (EVR)	0 (0-3)	0 (0-2)	0.690
Postoperative plasma transfusion (mL) (EVR)	0 (0-2100)	0 (0-400)	0.972
Postoperative hospital stays (day) (IQR)	7 (6-9.75)	8 (7-10)	0.175
Postoperative complications^1^			0.801
Grade I	5 (6.0%)	3 (3.6%)	
Grade II	8 (9.5%)	12 (14.3%)	
Grade IIIa	2 (2.4%)	1 (1.2%)	
Grade IIIb	0 (0.0%)	0 (0.0%)	
Grade IVa	2 (2.4%)	2 (2.4%)	
Grade IVb	0 (0.0%)	0 (0.0%)	
Grade V	0 (0.0%)	0 (0.0%)	
Liver failure^2^			1.000
Grade A	1 (1.2%)	1 (1.2%)	
Grade B	1 (1.2%)	1 (1.2%)	
Grade C	0 (0.0%)	0 (0.0%)	

^1^Postoperative complication is graded according to the Clavien-Dindo classification of surgical complications. ^2^Liver failure is graded according to the International Study Group of Liver Surgery (ISGLS) classification. Abbreviations: IQR: interquartile range; EVR: extreme value range; RBC: red blood cell.

**Table 4:** Uni- and multivariate analyses of disease-free survival (DFS) and overall survival (OS).

Variable	Number	Univariate		Multivariate	
		HR (95% CI)	*P* value	HR (95% CI)	*P* value
DFS					
Age, <60/≥60 y	84/84	1.562 (1.119–2.181)	0.009	1.590 (1.135–2.228)	0.007
Postoperative adjuvant TACE, no/yes	102/66	1.652 (1.176–2.320)	0.004	1.647 (1.170–2.320)	0.004
BCLC staging, B-C stage/A stage	137/31	1.293 (1.000–1.671)	0.050	1.288 (0.995–1.667)	0.055
LYM count, ≤1100/>1100/*μ*L	50/118	0.672 (0.507–0.890)	0.006	0.653 (0.484–0.880)	0.005
OS					
Age, <60/≥60 y	84/84	1.608 (1.110–2.328)	0.012	1.837 (1.259–2.680)	0.002
Reoperation, no/yes	153/15	2.313 (1.075–4.976)	0.032	1.647 (1.170–2.320)	0.011
GVI, yes/no	49/119	1.795 (1.217–2.648)	0.003	1.293 (0.670–2.494)	0.443
BCLC staging, B-C stage/A stage	137/31	1.543 (1.164–2.045)	0.003	1.349 (0.846–2.151)	0.209
LYM count, ≤1100/>1100/*μ*L	50/118	0.645 (0.467–0.891)	0.008	0.592 (0.419–0.838)	0.003

Abbreviations: HR: hazard ratio; CI: confidence interval.

## Data Availability

The data used to support the findings of this study are available from the corresponding author upon request.

## References

[1] Bray F., Ferlay J., Soerjomataram I., Siegel R. L., Torre L. A., Jemal A. (2018). Global cancer statistics 2018: GLOBOCAN estimates of incidence and mortality worldwide for 36 cancers in 185 countries. *CA: A Cancer Journal for Clinicians*.

[2] Forner A., Reig M., Bruix J. (2018). Hepatocellular carcinoma. *The Lancet*.

[3] Kluger M. D., Salceda J. A., Laurent A. (2015). Liver resection for hepatocellular carcinoma in 313 Western patients: tumor biology and underlying liver rather than tumor size drive prognosis. *Journal of Hepatology*.

[4] Bruix J., Gores G. J., Mazzaferro V. (2014). Hepatocellular carcinoma: clinical frontiers and perspectives. *Gut*.

[5] Sumie S., Kuromatsu R., Okuda K. (2008). Microvascular invasion in patients with hepatocellular carcinoma and its predictable clinicopathological factors. *Annals of Surgical Oncology*.

[6] Lim K. C., Chow P. K. H., Allen J. C. (2011). Microvascular invasion is a better predictor of tumor recurrence and overall survival following surgical resection for hepatocellular carcinoma compared to the Milan criteria. *Annals of Surgery*.

[7] Vauthey J. N., Lauwers G. Y., Esnaola N. F. (2002). Simplified staging for hepatocellular carcinoma. *Journal of Clinical Oncology*.

[8] Roayaie S., Blume I. N., Thung S. N. (2009). A system of classifying microvascular invasion to predict outcome after resection in patients with hepatocellular carcinoma. *Gastroenterology*.

[9] Rodríguez-Perálvarez M., Luong T. V., Andreana L., Meyer T., Dhillon A. P., Burroughs A. K. (2013). A systematic review of microvascular invasion in hepatocellular carcinoma: diagnostic and prognostic variability. *Annals of Surgical Oncology*.

[10] Xu L., Zhang M., Zheng X., Yi P., Lan C., Xu M. (2017). The circular RNA ciRS-7 (Cdr1as) acts as a risk factor of hepatic microvascular invasion in hepatocellular carcinoma. *Journal of Cancer Research and Clinical Oncology*.

[11] DeSantis C. E., Miller K. D., Dale W. (2019). Cancer statistics for adults aged 85 years and older, 2019. *CA: A Cancer Journal for Clinicians*.

[12] Asrani S. K., Devarbhavi H., Eaton J., Kamath P. S. (2019). Burden of liver diseases in the world. *Journal of Hepatology*.

[13] Su C. W., Lei H. J., Chau G. Y. (2012). The effect of age on the long-term prognosis of patients with hepatocellular carcinoma after resection surgery: a propensity score matching analysis. *Archives of surgery*.

[14] Cucchetti A., Sposito C., Pinna A. D. (2016). Effect of age on survival in patients undergoing resection of hepatocellular carcinoma. *The British Journal of Surgery*.

[15] Mirici-Cappa F., Gramenzi A., Santi V. (2010). Treatments for hepatocellular carcinoma in elderly patients are as effective as in younger patients: a 20-year multicentre experience. *Gut*.

[16] Mathers C. D., Stevens G. A., Boerma T., White R. A., Tobias M. I. (2015). Causes of international increases in older age life expectancy. *The Lancet*.

[17] Bruix J., Sherman M. (2011). Management of hepatocellular carcinoma: an update. *Hepatology*.

[18] Austin P. C. (2011). An introduction to propensity score methods for reducing the effects of confounding in observational studies. *Multivariate Behavioral Research*.

[19] Zhang W., Sun B. (2015). Impact of age on the survival of patients with liver cancer: an analysis of 27,255 patients in the SEER database. *Oncotarget*.

[20] Xu X. S., Chen W., Miao R. C. (2015). Survival analysis of hepatocellular carcinoma: a comparison between young patients and aged patients. *Chinese Medical Journal*.

[21] Nomura F., Ohnishi K., Honda M., Satomura Y., Nakai T., Okuda K. (1994). Clinical features of hepatocellular carcinoma in the elderly: a study of 91 patients older than 70 years. *British Journal of Cancer*.

[22] Namieno T., Kawata A., Sato N., Kondo Y., Uchino J. (1995). Age-related, different clinicopathologic features of hepatocellular carcinoma patients. *Annals of Surgery*.

[23] Katsuta E., Tanaka S., Mogushi K. (2014). Age-related clinicopathologic and molecular features of patients receiving curative hepatectomy for hepatocellular carcinoma. *American Journal of Surgery*.

[24] Ang C., Shields A., Xiu J. (2017). Molecular characteristics of hepatocellular carcinomas from different age groups. *Oncotarget*.

[25] Steelman L. S., Chappell W. H., Abrams S. L. (2011). Roles of the Raf/MEK/ERK and PI3K/PTEN/Akt/mTOR pathways in controlling growth and sensitivity to therapy-implications for cancer and aging. *Aging*.

[26] Zhang H., Li X. X., Yang Y., Zhang Y., Wang H. Y., Zheng X. F. S. (2018). Significance and mechanism of androgen receptor overexpression and androgen receptor/mechanistic target of rapamycin cross-talk in hepatocellular carcinoma. *Hepatology*.

[27] Kalra M., Mayes J., Assefa S., Kaul A. K., Kaul R. (2008). Role of sex steroid receptors in pathobiology of hepatocellular carcinoma. *World Journal of Gastroenterology*.

[28] Vivanco I., Sawyers C. L. (2002). The phosphatidylinositol 3-kinase-AKT pathway in human cancer. *Nature Reviews Cancer*.

[29] Chen C. H., Chang T. T., Cheng K. S. (2006). Do young hepatocellular carcinoma patients have worse prognosis? The paradox of age as a prognostic factor in the survival of hepatocellular carcinoma patients. *Liver International*.

[30] Saneto H., Kobayashi M., Kawamura Y. (2008). Clinicopathological features, background liver disease, and survival analysis of HCV-positive patients with hepatocellular carcinoma: differences between young and elderly patients. *Journal of Gastroenterology*.

[31] Du M., Chen L., Zhao J. (2014). Microvascular invasion (MVI) is a poorer prognostic predictor for small hepatocellular carcinoma. *BMC Cancer*.

[32] Yamashita Y., Tsuijita E., Takeishi K. (2012). Predictors for microinvasion of small hepatocellular carcinoma </= 2 cm. *Annals of Surgical Oncology*.

[33] Global Burden of Disease Cancer Collaboration, Fitzmaurice C., Akinyemiju T. F. (2018). Global, regional, and national cancer incidence, mortality, years of life lost, years lived with disability, and disability-adjusted life-years for 29 Cancer Groups, 1990 to 2016: a systematic analysis for the global burden of disease study. *JAMA Oncology*.

[34] Hori M., Tanaka M., Ando E. (2014). Long-term outcome of elderly patients (75 years or older) with hepatocellular carcinoma. *Hepatology Research*.

[35] Trevisani F., D'Intino P. E., Grazi G. L. (1996). Clinical and pathologic features of hepatocellular carcinoma in young and older Italian patients. *Cancer*.

[36] Oishi K., Itamoto T., Kobayashi T. (2009). Hepatectomy for hepatocellular carcinoma in elderly patients aged 75 years or more. *Journal of Gastrointestinal Surgery*.

